# Surfactant addition in diesel oil degradation – how can it help the microbes?

**DOI:** 10.1007/s40201-020-00494-9

**Published:** 2020-06-20

**Authors:** Agata Zdarta, Wojciech Smułek, Amanda Pacholak, Beata Dudzińska-Bajorek, Ewa Kaczorek

**Affiliations:** 1 Institute of Chemical Technology and Engineering, Poznan University of Technology, Berdychowo 4, 60-965 Poznan, Poland; 2 Hipolit Cegielski State College of Higher Education in Gniezno, Gniezno, Poland

**Keywords:** Surfactant, Biodegradation, Diesel oil, Microorganisms’ properties, Genetic changes, Toxicity

## Abstract

**Purpose:**

Despite wide research on bioremediation of hydrocarbon-contaminated soil, the mechanisms of surfactant-enhanced bioavailability of the contaminants are still unclear. The presented study was focused on the in-depth description of relationships between hydrocarbons, bacteria, and surfactants. In order to that, the biodegradation experiments and cell viability measurements were conducted, and the properties of cell surface were characterized.

**Methods:**

MTT assay was employed to measure plant extracts toxicity to microbes. Then, membrane permeability changes were evaluated, followed by diesel oil biodegradation in the presence of surfactants measurements by GCxGC-TOFMS and PCR-RAPD analysis.

**Results:**

Our study undoubtedly proves that different surfactants promote assimilation of different groups of hydrocarbons and modify cell surface properties in different ways. Increased biodegradation of diesel oil was observed when cultures with *Acinetobacter calcoaceticus* M1B were supplemented with *Saponaria officinalis* and *Verbascum nigrum* extracts. Interestingly, these surfactants exhibit different influences on cell surface properties and their viability in contrast to the other surfactants. Moreover, the preliminary analyses have shown changes in the genome caused by exposure to surfactants.

**Conclusions:**

The results indicated that the benefits of surfactant use may be related to deep modification at the omics level, not only that of cell surface properties and confirms the complexity of the interactions between bacterial cells, pollutants and surfactants.

**Electronic supplementary material:**

The online version of this article (10.1007/s40201-020-00494-9) contains supplementary material, which is available to authorized users.

## Introduction

Soil and water contamination by petroleum-based hydrocarbons is one of the most acute environmental problems. The main sources of pollution are accidental oil spills during exploitation, transport, processing and storage, drilling operations and improper discharge of oil products into the environment. Diesel oil and its components have been considered as emerging pollutants which display the negative, toxic impact on humans, other living organisms and microorganisms in the environment [1–3]. The contaminated ecosystems are able to regenerate themselves, however, these processes are relatively slow due to an inadequate amount of nutrients, oxygen, and microorganisms capable of biodegradation. Therefore, investigation of new treatment strategies is essential. Conventional removal methods are expensive and may produce toxic residues. An important way of recovering soil from contaminated areas, causing minimal damage to the total environment, is efficient and low-cost microbial bioremediation [1, 4, 5]. Nevertheless, the biodegradation process by pure microbial cultures present in the ecosystems might be insufficient. Hence, many factors have been investigated to enhance biodegradation effectiveness. They include, among others, the use of mixed bacterial cultures [6], addition of chemical oxidants [5], surfactants of both natural and synthetic origin [1, 2, 7–10] or other factors promoting the reduction of hydrocarbon droplets and the growth of microorganisms [11, 12]. However, it is worth noticing that the introduction of additional components to microbial cultures makes the phenomenon of biodegradation very complex. When designing and optimizing bioremediation process, such factors as pH, temperature, number of microorganisms and the presence of an additional component in microbial culture must be taken into account. These factors not only affect biodegradation efficiency but also microbial cell properties [13], changes in bacterial gene material [14] or adverse changes in physicochemical soil and water properties [11].

Although the biodegradation of diesel oil and its derivatives has been thoroughly investigated in recent years, this topic remains of top interest and is not fully understood [11, 15–18]. Although surfactants-enhanced biodegradation of hydrocarbon has been studied for years, comprehensive analysis of this process, taking into account multi-level changes occurring in cells - from the genome, through metabolic activity, to surface properties of cells, is still lacking. Results of the presented research can significantly complement the hitherto knowledge on this subject. Hence, the aim of this study was to examine the impact of diesel oil and selected surface-active agents on bacterial cell properties and changes in their gene material. Moreover, biodegradation of total diesel oil hydrocarbons, its aliphatic and aromatic fraction has been investigated.

## Materials and methods

### Chemicals

All chemicals used in the experiments were of analytical grade. Diesel oil was purchased from petroleum station (PKN Orlen); reference surfactants Rhamnolipid JBR 425 and Triton X-100 were purchased from the manufacturers (Jeneil Biosurfactants Co. and Sigma-Aldrich, respectively); 3-(4,5-Dimethylthiazol-2-yl)-2,5-Diphenyltetrazolium Bromide (MTT), 2-Nitrophenyl *β*-D-galactopyranoside (ONPG), methanol, and hexane were purchased from Sigma-Aldrich (Poland).

### Surfactants

Surface active compounds rich extracts were obtained from *Aesculus hippocastanum* bark, *Verbascum nigrum* flowers, *Saponaria officinalis* roots and *Sapindus mukorossi* nuts. Respective dry plant fragments, in form provided by the supplier (FLOS, Poland), were placed in cellulose extraction tubes and extracted for 8 h at 65 °C with methanol as a liquid phase. The solvent was evaporated (Büchi vapor) and the extracts were freeze-dried (Alpha 1–2 LD plus, Christ, Germany). Afterward, obtained lyophilizes, Rhamnolipid JBR 425 and Triton X-100 were solved in MiliQ water to obtain final concentration equal to 10 CMC (critical micelle concentration), pasteurized (65 °C, 25 min) and filtrated via 0.2 μm filter before use. Table 1 presents the origin and CMC value of each surfactant used.

**Table 1:** Surfactants origin and CMC values

Surfactant	Origin	CMC value (g/L)	Reference
*Aesculus hippocastanum* bark	plant	0.85	[19]
*Verbascum nigrum* flowers	plant	10.0	[20]
*Saponaria officinalis* roots	plant	0.95	[21]
*Sapindus mukorossi* nuts	plant	0.10	[22]
Rhamnolipid JBR 425	bacterial	0.12	Safety data sheet Jeneil Biosurfactants
Triton X-100	synthetic	0.19	Safety data sheet Sigma-Aldrich

### Bacterial isolation and cultivation

Soil samples were collected from long-term hydrocarbon contaminated soil [23] and the isolated bacteria were identified using biochemical (Vitek® 2 system, Biomerieux) and molecular techniques. Their nucleotide sequences are available in GenBank under the accession numbers: KX.667738.1 (*Raultella planticola* M01) and KU.563543.1 (*Acinetobacter calcoaceticus* M1B). Selected results of biochemical profiling obtained using Vitek® 2 system (Biomerieux, Poland) are presented in Supplementary Materials 1 (S1). The pure bacterial strains were stored on nutrition agar plates (Biomerieux, Poland).

Cells cultivation was performed using mineral salts medium (MSM) and trace elements solution as described in [24]. For microbial experiments cells inocula of each strain were prepared by adding 50 mL of MSM medium, 150 μL of trace elements solution, 1 mL of 20% sodium succinate and loop-full of bacterial cells from agar plate to 250 mL glass bottle. After 24 h incubation (30 °C) culture optical density was verified spectrophotometrically (OD_550_ ~ 1.0 for diesel oil cultures) and 1 mL of inocula was used for diesel oil cultures preparation. Diesel oil cultures were prepared by mixing 18 mL of MSM medium, 60 μL of trace elements solution, 200 μL od diesel oil (DO), 2 mL of bacterial cells inocula and surfactant in an amount equal to obtain a final concentration of 1 CMC in the culture.

20 mL diesel oil cultures were incubated in 100 mL Duran Schott glass bottles at 30 °C, with shaking (120 rpm) for 14 days. Similar test systems were carried out for genetic analysis with selected surfactants (*S. mukorossi* and *S. officinalis*). Each experimental set was made in triplicate.

### Cells surface properties

Cells from inocula were centrifuged (4000 rpm, 10 min) and washed trice with sterile MSM medium. Afterward, cells were diluted to optical density OD_550_ ~ 0.6–0.9 and investigated with a view to metabolic activity (with MTT test) and cell membrane permeability (MP test) using the methods described earlier [25]. Metabolic activity measurements experiments were performed in 1.5 mL Eppendorf tubes, and the experimental set up consisted of 30 μL of MTT reagent, 500 μL of bacterial suspension, surfactant in an amount equal to obtain a final concentration corresponding to 1, 2 or 3 CMC and MSM medium to a final volume of the sample of 800 μL. Membrane permeability experiments were performed in similar experimental setups as MTT tests, although instead of MTT reagent 25 μL of ONPG reagent was added. Each experiment was made in triplicate and the mean values of the spectrophotometric measurements are presented.

### Diesel oil biodegradation

The diesel oil concentration in samples was determined using the method described by [24] (2018) with some modifications. Briefly, after cultures extraction with hexane, the hydrocarbons were identified and quantitatively analyzed using GC-MS/MS chromatograph (Pegasus 4D, GCxGC-TOMFMS, LECO, St Joseph, MI, USA). After splitless injection of 1 μL of the sample, the oven temperature was set at 40 °C, maintained for 2 min, then heated up to 300 °C at a rate of 10 °C min^−1^ and maintaining the temperature of the oven for 15 min. The quantitative analysis for total, aliphatic and monoaromatic hydrocarbons were conducted on the base of the calibration curve and qualitative identification of compounds using mass spectra.

### Genetic analysis

For genetic analysis, bacteria cultivated on medium with diesel oil were centrifuged and the DNA was isolated using a commercial DNA kit (Gene Elute Bacterial DNA kit, Sigma-Aldrich, Poland). Random amplification of polymorphic DNA (RAPD) was performed by the use of 80 arbitrary primers. The discriminatory primers were: A1 (5’ CAGGCCCTTC 3′); A2 (5’ TGCCGAGCTG 3′); A3 (5’ AGTCAGCCAC 3′) and the analysis were performed according to the method described earlier [26].

## Results

### Impact of analyzed surfactants on cells properties

**Fig. 1:**
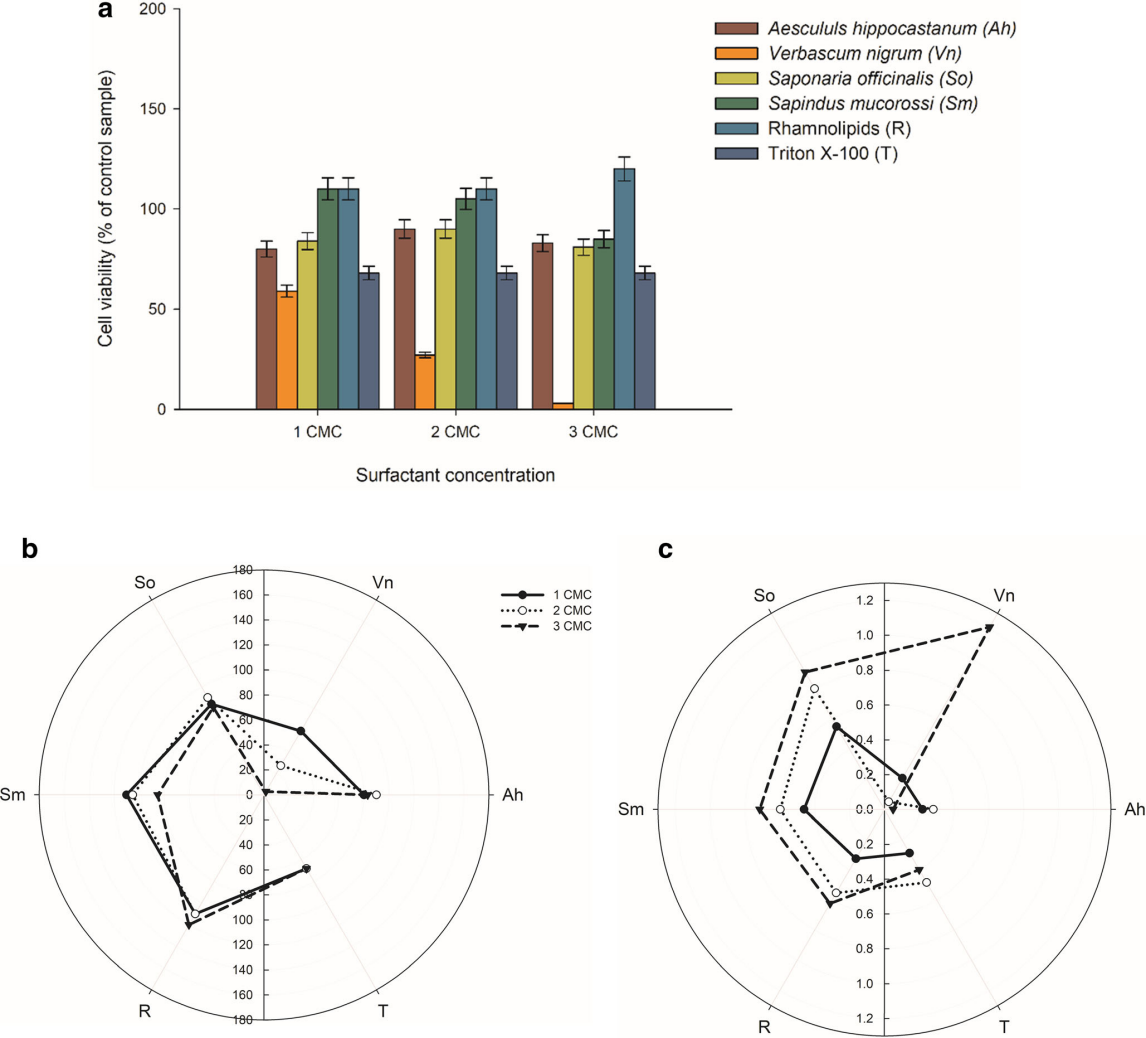
*R. planticola* M01 cells viability in cultures with surfactants addition in different concentrations (**a**), polar plots of examined surfactants toxicity (**b**) and impact on cell inner membrane permeability (**c**) in analyzed concentrations

The most sensitive to the natural surfactants among all studied cells was strain *R. planticola* M01 (Fig. 1a). Simultaneously, the metabolic activity of its cells was the highest of all with three examined Triton X-100 concentrations. Moreover, the cells were very sensitive to *Verbascum nigrum* extract, especially at high concentration. On the other hand, the addition of *Sapindus mukorossi* extract and rhamnolipids in concentrations equal to 1 or 2 CMC value, boosted their viability over the level of glucose-cultured cells.

When it comes to the results of membrane permeability measurements, at a concentration of plant extracts equal to 1 CMC, statistically significant differences were observed for the *R. planticola* M01 cells grown the extracts (Table 2). The inner membrane permeability increased with each increase in the extracts concentrations. At the highest concentration of the extracts, the cells were characterized by low viability and very high membrane permeability (Fig. 1b and c). The most pronounced differences in viability were observed when *V. nigrum* and *S. officinalis* were used. From among the extracts studied, the impact of those from *A. hippocastanum* on the cells was the lowest as the differences in cells viability and membrane permeability were almost unnoticeable. For the plant extracts and rhamnolipids, the cell membrane permeability increased with increasing surfactant concentration. Only for the cultures with Triton X-100, the highest permeability was observed at 2 CMC.

**Table 2:** Membrane permeability of *R. planticola* M01 cells cultured with different surface active agents concentrations

Membrane permeability (μM ONP/min)			
Surface active agent	Surface active agent concentration		
	1 CMC	2 CMC	3 CMC
*A. hippocastanum*	0.22 ± 0.01	0.28 ± 0.01	0.05 ± 0.01
*V. nigrum*	0.21 ± 0.01	0.05 ± 0.01	1.21 ± 0.06
*S. officinalis*	0.55 ± 0.03	0.80 ± 0.04	0.91 ± 0.05
*S. mucorrosi*	0.46 ± 0.02	0.60 ± 0.03	0.72 ± 0.04
Rhamnolipids	0.33 ± 0.02	0.55 ± 0.03	0.62 ± 0.03
Triton X-100	0.29 ± 0.01	0.48 ± 0.02	0.40 ± 0.02

**Fig. 2:**
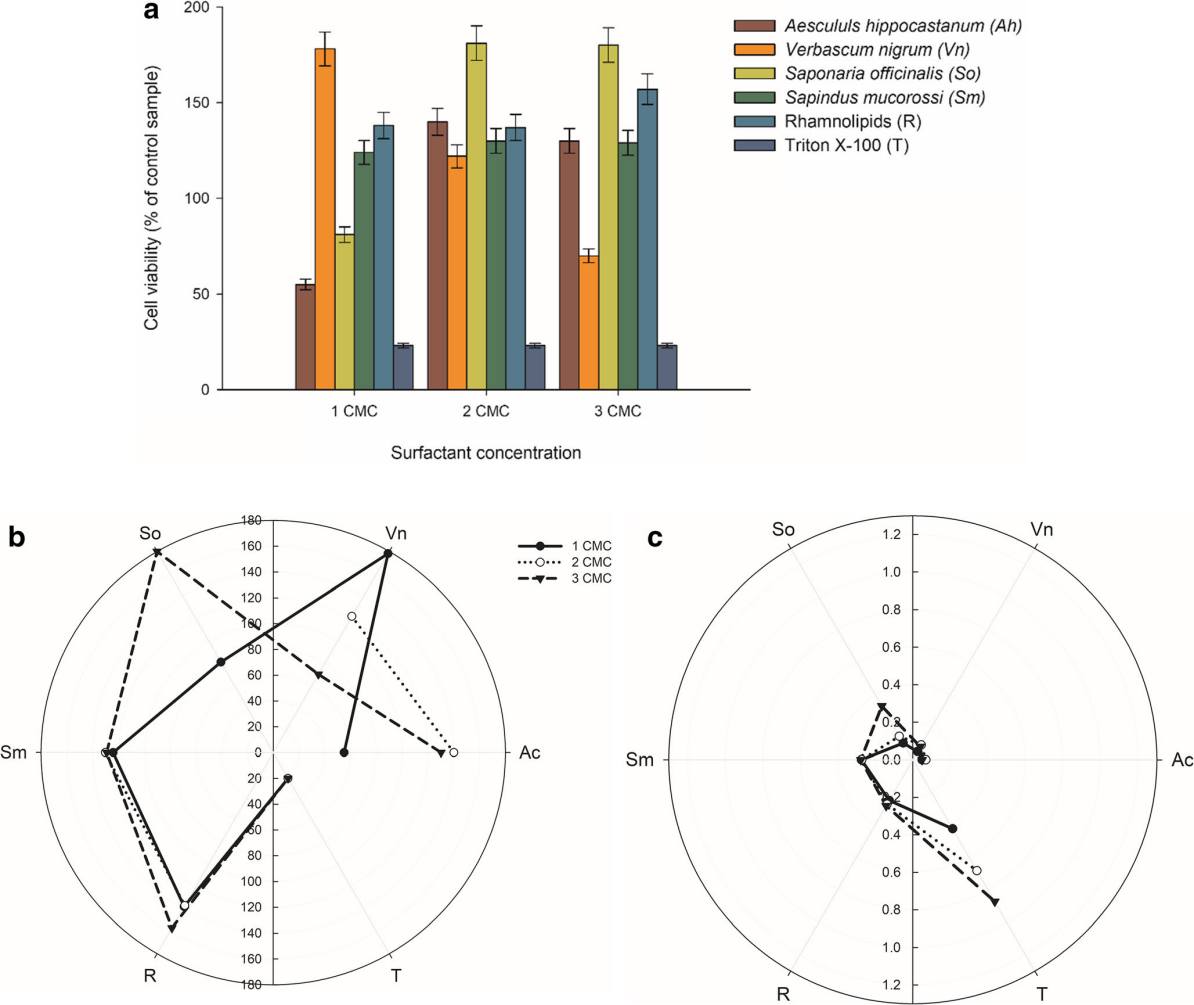
*A. calcoaceticus* M1B cells viability in cultures with surfactants addition in different concentrations (**a**), polar plots of examined surfactants toxicity (**b**) and impact on cell inner membrane permeability (**c**) in analyzed concentrations

It should be emphasized that the strain *A. calcoaceticus* M1B reacted differently from the *Raultella* strain. Increasing concentrations of *A. hippocastanum*, *S. officinalis*, *S. mukorossi,* and rhamnolipids were favorable for these microorganisms, and the highest metabolic activity of the cells was observed in the cultures with surfactant content equal to 2 and 3 CMC of *S. officinalis* added (Fig. 2a). Surprisingly, the cells were also very active in the presence of 1 CMC of *Verbascum nigrum* extract added, but for this surfactant the cells viability dropped significantly with increasing surfactant concentration. It is also worth mentioning that *A. calcoaceticus* M1B viability was the most obstructed in the Triton X-100 presence in the culture.

The response of *Acinetobacter calcoaceticus* M1B cells membrane to the contact with surfactants is shown in Fig. 2c and Table 3. In the systems with Triton X-100, the cell membrane permeability increased above the value obtained for the control sample, which was consistent with decreasing cells metabolic activity in these systems (Fig. 2b and c). In the experiment testing the effect of plant extracts and rhamnolipids, the permeability of the cell membrane was low and did not significantly differ from the cell membrane permeability in the reference sample. For this strain, membrane permeability test results were the lowest and reflected the high cells viability in the presence of the analyzed natural surfactants in all concentrations.

**Table 3:** Membrane permeability of *A. calcoaceticus* M1B cells cultured with different surface active agents concentrations

Membrane permeability (μM ONP/min)			
Surface active agent	Surface active agent concentration		
	1 CMC	2 CMC	3 CMC
*A. hippocastanum*	0.05 ± 0.00	0.07 ± 0.01	0.05 ± 0.01
*V. nigrum*	0.05 ± 0.00	0.09 ± 0.01	0.08 ± 0.01
*S. officinalis*	0.10 ± 0.01	0.14 ± 0.01	0.33 ± 0.02
*S. mucorrosi*	0.27 ± 0.01	0.27 ± 0.01	0.28 ± 0.01
Rhamnolipids	0.25 ± 0.01	0.27 ± 0.01	0.28 ± 0.01
Triton X-100	0.42 ± 0.02	0.68 ± 0.03	0.87 ± 0.04

### Diesel oil hydrocarbons biodegradation

**Fig. 3:**
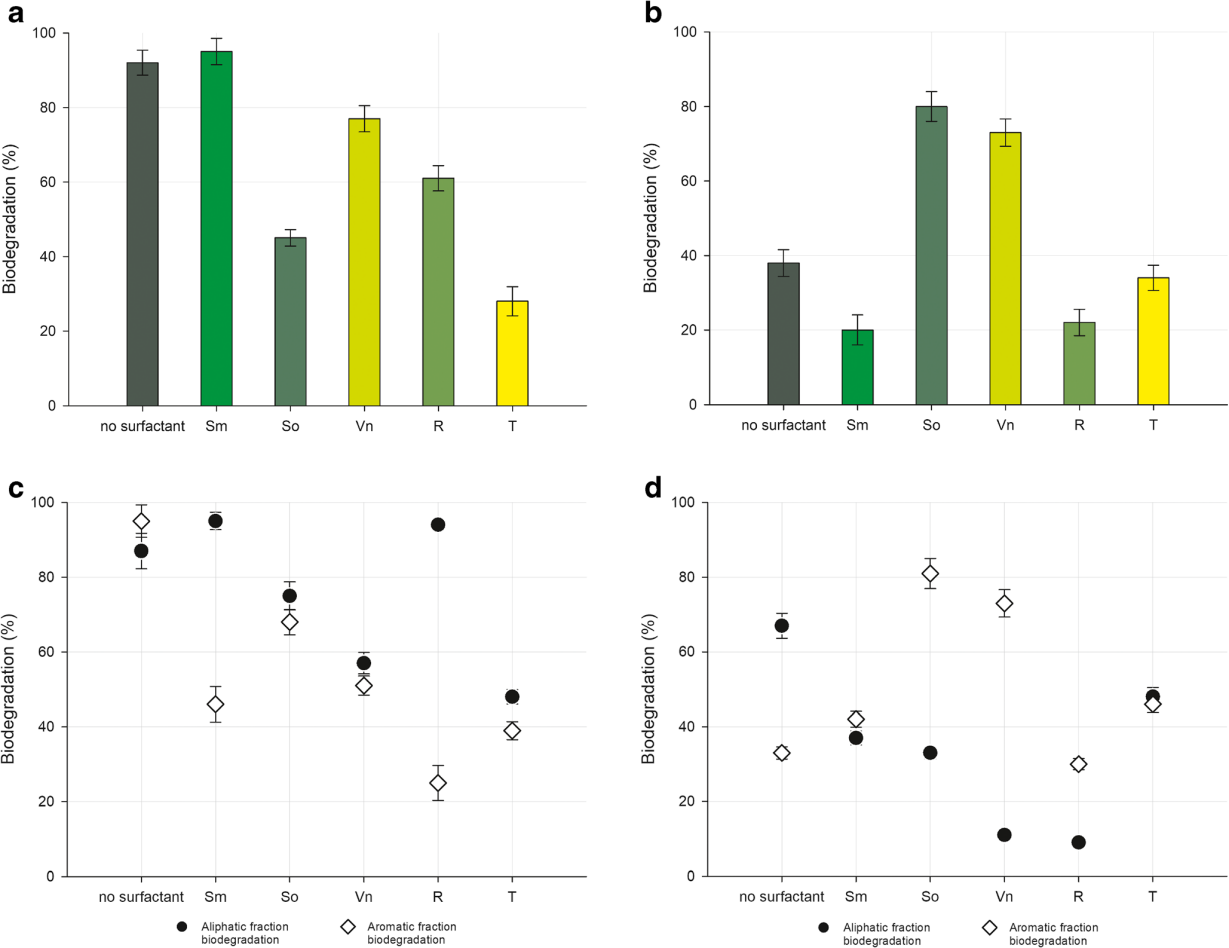
Diesel oil total hydrocarbons (**a, b**), and aliphatic (●) and monoaromatic (◊) fractions biodegradation (**c, d**) after 14 days by *R. planticola* M01(**a, c**) and *A. calcoaceticus* M1B (**b, d**) cultures without any surfactant (**no surfactant**) or with surfactants: *S. mukorossi* extract (**Sm**); *S. officinalis* extract (**So**); *V. nigrum* extract (**Vn**) *Pseudomonas aeruginosa* rhamnolipids (**R**) or Triton X-100 (**T**)

According to the results of diesel oil biodegradation, evaluated on the basis of the total hydrocarbons content after 14 days of the process, *R. planticola* M01 strain showed the highest biodegradation potential as it degraded 92% of the diesel oil (Fig. 3a) in this time. In a similar period, *A. calcoaceticus* M1B strain biodegraded 38% of diesel oil. Interestingly, apart from the addition of *S. mukorossi* extract to *R. planticola* M01 cells, the addition of the other surfactants did not improve the biodegradation efficiency (Fig. 3).

For *A. calcoaceticus* M1B samples, the presence of the surfactants caused a decrease in the diesel oil biodegradation (Fig. 3b). The most significant decrease in the total hydrocarbons’ degradation efficiency was noted when *R. planticola* M01 cells were cultured with Triton X-100 addition (Fig. 3a). It is also worth mentioning that the lowest observed values of total diesel oil hydrocarbons removal were observed for *A. calcoaceticus* M1B strain.

In order to assess the removal of different fractions of diesel oil, extensive analysis of the obtained results was performed, to evaluate the amounts of aliphatic and monoaromatic fractions degraded in the analyzed samples. As follows from the results obtained for the aliphatic fraction of diesel oil (Fig. 3d), a decrease in biodegradation efficiency in the presence of surfactants was observed for *A. calcoaceticus* M1B strain. In the presence of natural surfactants, the decrease in the removal of aliphatics was as big as ~40% for *S. mukorossi* extract and reached even ~80% for rhamnolipids. Such a strong impact was not observed for Triton X-100. What is interesting, the opposite response was noted for *R. planticola* M01 cells (Fig. 3c). The addition of natural surfactants slightly increased the degradation of aliphatics by this strain but caused a sharp decrease in this degradation when Triton X-100 was added. Furthermore, the results obtained for aromatic compounds show a decrease in monoaromatic hydrocarbons removal with natural surfactants addition and the most prominent changes were observed for rhamnolipids. In contrast, for *A. calcoaceticus* M1B the addition of surfactants increased the degradation of monoaromatics, which was the most pronounced in the cultures with *S. officinalis* and *S. mukorossi* extracts. However, similarly as for *A. calcoaceticus* M1B strain, rhamnolipids were the least effective (Fig. 3d). It should be indicated that the plant extracts showing surfactant properties exhibited significantly stronger impact on biodegradation efficiency than the other tested surfactants.

### Genetic modifications of the cells

**Fig. 4:**
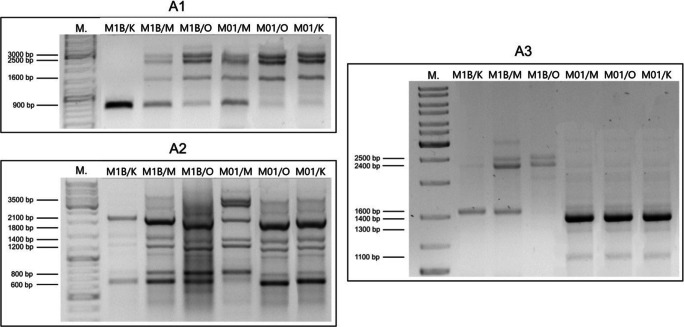
RAPD-PCR patterns generated by *A. calcoaceticus* M1B and *R. planticola* M01 using A1, A2 and A3 primers: **M** – 100 bp PCR DNA marker; **M1B/K** – control sample of M1B strain; **M1B/M** – M1B strain cultivated with *S. officinalis* extract; **M1B/O** – M1B strain cultivated with *S. mukorossi* extract; **M01/K** – control sample of M01 strain; **M01/M** – M01 strain cultivated with *S. officinalis* extract; **M01/O** – M01 strain cultivated with *S. mukorossi* extract

Preliminary analysis of genetic modifications revealed significant changes in genetic sequences of surfactant-exposed cells. As the extracts from *S mukorossi* and *S. officinalis* were clear and colorless, besides their extraction process showed higher reproducibility, these extracts were selected for these tests. Furthermore, they are characterized by different CMC values and significant differences between these two extracts were observed in the above-described research. Here, we present selected results for both analyzed strains, after contact with *S. mukorossi* and *S. officinalis* extracts (Fig. 4).

The used primers were differentiating in all three analyzed cases for *A. calcoaceticus* M1B strain, and in two cases for *R. planticola* M01 strain. These primers refer to oxygenase genes in *Pseudomonas* spp. [27] and should be complimentary to oxygenase genes or gene fragments from the two used strains, as the genes responsible for oxygenase synthesis differ slightly between the bacterial strains [28]. In our study *A. calcoaceticus* M1B reacted similarly in the presence of both plant surfactants added, but the changes induced by *S. officinalis* were more pronounced. Moreover, there were additional bands visible in the images after electrophoresis recorded in the presence of this surfactant when primer A3 was used (over 3000 bp). This surfactant was also more effective for *R. planticola* M01 cells with an additional pattern visible when A1 primer was used, which was significantly different from the pattern obtained for the other two samples with A2 primer. It is worth noting that the bands obtained with the use of the *S. officinalis* extract made a similar pattern for the two tested strains (with A1 and A2 primers). A similar effect can be observed with the same primers in test systems with *S. mukorossi* extract addition. This suggests the analogous influence of saponins-containing extracts on the genetic material of different microbial strains. Although genetic modifications induced by the extracts might have comparable character, the morphological effect on the cells was significantly different. The presence of the two extracts led to different results in cell metabolic activity and membrane permeability tests as well as in the biodegradation test. Interestingly, the addition of *S. officinalis* extract to *R. planticola* M01 cultures resulted in lower viability of the cells, higher membrane permeability and a higher level of DO total and greater monoaromatic hydrocarbons biodegradation. A similar amount of this extract induced high viability of *A. calcoaceticus* M1B cells, characterized by low membrane permeability and lower DO total hydrocarbons biodegradation.

## Discussion

Increasing interest in surfactants application for environment cleanup raises the question about their impact on natural microflora in comparison to that of the petroleum-derived surfactants. To analyze this problem, we performed MTT analysis, evaluating the impact of different surfactants on *R. planticola* M01 and *A. calcoaceticus* M1B cells metabolic activity (Figs. 1, 2, 3 and 4). The MTT assay is a colorimetric analysis, widely used for cells metabolic activity assessment. The assay is fast and simple to perform, relying on the reduction of yellow water-soluble tetrazolium dye to purple formazan crystals, by mitochondrial dehydrogenases, reflecting the number of viable cells present in the culture. From the obtained results it can be seen that the natural surfactants: rhamnolipids and plant extracts have the most favorable effect on bacterial cells. Viability of the cells exceeded 100% in all samples containing rhamnolipids and in eight from nine samples with *S. mukorossi* extract addition. The third substance according to the improving effect on cells activity was *S. officinalis* extract, with cells viability over 100% in five cases. Moreover, in none of the samples with the three above-mentioned surfactants, the cytotoxic effect of the surface active compounds was observed. In accordance with the present results, previous studies have demonstrated that the analyzed impact of *Glandula riaselloi* leaves extract has no negative effect on Vero cells until its concentration exceeds 10 μg/mL [29] (Figueiró et al. 2016). On the other hand, the most invading was Triton X-100. In none of the presented samples containing this surface active agent, the viability of the cells exceeds 70%. Moreover, in four cases of nine, Triton X-100 caused a decrease in cells metabolic activity below 30%, with the lowest values observed for *A. calcoaceticus* M1B. This surface active compound is a nonionic detergent, widely used worldwide as a noninvasive and non-toxic one. However, there are multiple papers describing its harmful effect on microorganisms and cell lines [30–32].

As to the influence of *A. hippocastanum* and *V. nigrum* on the analyzed strains, it is worth mentioning that only these two extracts were dark orange-brown colored. While MTT assay uses spectrophotometric measurements to determine cells viability, the tone of the extract might have a significant impact on the results of the test [33]. Furthermore, the MTT assay results might be falsely positive, due to accidental removal of the analyzed cells during a wash in 1-isopropanol solution [34–36]. Formazan crystals formed during the reaction might also disrupt cell membranes, resulting in false negative outcomes [37]. [3-(4,5-dimethylthiazol-2-yl)-2,5-diphenyltetrazolium bromide], the main component of the MTT assay is also sensitive to light [34].

This inconsistency of results obtained in the presence of plant extracts may be due to the lack of their additional purification, besides filtration, as the presence of other active compounds might influence the obtained results. Substances such as ascorbic acid, coenzyme A (CoA), or compounds containing thiol groups can reduce tetrazolium salts leading to enhanced absorbance in the samples [34, 36]. Therefore, the results of MTT assay performed with raw plant extracts need to be interpreted with caution, as many factors can affect the final result. The best solution is the performance of additional tests for detailed study of extracts impact on the bacterial cells. For this reason, the ONPG assay was implemented to determine plant extract-cell membrane interactions.

Analysis of the results of extracts toxicity studies prompted us to undertake a study of the surfactants impact on the bacteria cells’ membranes. The amphiphilic properties of the surfactants allow them to penetrate the phospholipids layer. Moreover, the surface active compounds may modify the structure of the proteins responsible for transport in the cell membrane. Therefore, we performed inner cell membrane permeability test (ONPG assay), to explore the impact of selected surface active extract on the bacterial strains membrane integrity. The collected results indicated strong effect of the surfactants used on bacterial membrane permeability. The amphiphilic properties of the surfactants strongly influence the cell membrane, which is frequently responsible for antimicrobial properties of the biosurfactants [38]. However, [39] have observed that saponins from *Quillaja saponaria* increased the membrane permeability of clinical *Escherichia coli* strain without affecting its survival. It can be crucial for effective biodegradation of hydrophobic pollutants because the limited permeability of the cell envelope can also limit the transmembrane transport, which may be the rate-limiting step in hydrocarbons utilization. Moreover, the surfactants, as permeabilizing agents, can increase the transmembrane diffusion of the pollutants [40]. Taking into account relatively high toxicity of *A. hippocastanum* and *V. nigrum* towards *A. calcoaceticus* M1B cells, as well as the positive effect of *S. muccorosi* and rhamnolipids on all analyzed strains, the two later extracts were used for further analysis, together with the synthetic surfactant Triton X-100.

Analysis of biodegradation of all hydrocarbons and the dominating fractions in diesel fuel, the aliphatics and monoaromatics, revealed the difference between the tested microorganisms. On the one hand, the best biodegrading strain of all fractions was *R. planticola* M01. On the other hand, *A. calcoaceticus* M1B definitely prefers the aliphatic than monoaromatic hydrocarbons. The impact of surfactants on biodegradation of hydrophobic hydrocarbons has been widely discussed, however, the complexity of the issue still needs new and valuable data. What is more, the positive or negative effect of the addition of surfactants strongly depends on the kind of tested compound as well as on bacterial strain. Smułek et al. (2016) have noticed that *S. mukorossi* extract can improve biodegradation of the diesel oil, but only by the microorganisms not exposed to the hydrocarbon pollutants previously. The explanation of the reduction of biodegradation of hydrophobic pollutants in the presence of surfactants has been proposed by [41]. They indicated that the hydrocarbons bioavailability reduction may be caused by blocking the effect of rhamnolipid molecule layer at the organic-water interface. However, the rhamnolipids can modify cell surface hydrophobicity and as a result enhance the biodegradation of non-aqueous pollutants [42].

A significant novelty of this study is the performance of genetic studies of the cells exposed to natural surfactants. The literature information about genetic modifications in bacteria induced by contact with saponins-containing plant extracts is scarce. The overly-prescriptive information concerns only genotoxicity of this agents, concerning *Quillaja* saponins (as an E999 food additive) [43], *Anagallis arvensis* L. extract effect on *Candida albicans* strains [44] and *Pityrocarpa moniliformis* extract genotoxicity in micronucleus test [44]. All the above-mentioned papers have shown no genotoxic effect on the cells. The EFSA report has shown no mutagenic effect of saponins in concentrations up to 5 μL per plate in four strains of *Salmonella typhimurium* (TA1535, TA1537, TA98 and TA100) and in one of *E. coli* (strain WP2 uvrA). Our previous study revealed significant differences in the genetic material of *Rahnella* sp. EK12 strain induced by *Quillaja* saponins and rhamnolipids [26]. The changes that we have observed in cell genetic profile are the evidence of deep modifications of cells properties induced by the surfactants, but do not influence significantly cells viability, rather promoting selected hydrocarbons degradation.

## Conclusions

The collected results show a complex relationship between the surfactants presence and assimilation of hydrocarbons by bacteria. Increased biodegradation of diesel oil was observed when the culture with *A. calcoaceticus* M1B was supplemented with *S. officinalis* and *V. nigrum* extracts. In our study *A. calcoaceticus,* M1B reacted similarly for both plant surfactants added, but *S. officinalis* extract induced genomic changes, which were more pronounced. Moreover, there are additional patterns visible in PCR products after electrophoresis of samples with this surfactant while primer A3 was used (over 3000 bp). Interestingly, different surfactants promote assimilation of different groups of hydrocarbons and have modified cell surface properties in different ways. This observation confirms the complexity of the interactions between bacteria cells, pollutants, and surfactants, influencing both bacteria genome and cell outer structure.

## Electronic supplementary material

ESM 1 (DOCX 17 kb)
