# Supplementary material for: Surfactant addition in diesel oil degradation – how can it help the microbes?
Source: J Environ Health Sci Eng. 2020 Jun 20;18(2):677–86. doi: 10.1007/s40201-020-00494-9 (PMC7721782; doi:10.1007/s40201-020-00494-9)
Supplement: Supplementary file 1 — (DOCX 17 kb) [file 40201_2020_494_MOESM1_ESM.docx]

**Surfactant addition in diesel oil degradation – how can it help the microbes?**

***Journal: Environmental Science and Pollution Research***

Agata Zdarta*^1^ (ORCID 0000-0003-2835-5447), Wojciech Smułek^1^ (ORCID 0000-0001-5377-9933), Amanda Pacholak^1^ (ORCID 0000-0001-9520-4361), Beata Dudzińska-Bajorek^2^,
Ewa Kaczorek^1^ (ORCID 0000-0003-2478-4738)

^1^ Institute of Chemical Technology and Engineering, Poznan University of Technology, Berdychowo 4, 60-965 Poznan, Poland; ^2^ Hipolit Cegielski State College of Higher Education in Gniezno, Poland

*corresponding author: agata.zdarta@doctorate.put.poznan.pl; phone: +48 61 665 36 86; fax:+48 61 665 28 52

S1.

*Biochemical test*

Biochemical heterogeneity is a consequence of metabolic specialization of the cells and its flexibility to adapt to the uptake of a specific metabolite. Presented in Table 1 results clearly show similarities and differences in the phenotypes of the selected strains.

Each of the tested strains has specific properties, distinguishing it from the others:
*R. planticola* M01 is capable of the production of Glycine arylamidase and
γ-glutamyl-transferase; while *A. genomospecies* M01 ferment D-mannitol and D-cellobiose and assimilate L-histidine. Moreover, *R. planticola* M01 has three similarities with *A. genomospecies* M1B in alkalization of L-lactate and coumarate utilization and are resistant to O/129 (comp. vibrio). Obtained metabolic fingerprints for the selected organisms led us to their identification as *Pseudomonas fluorescens* M01 (95%); and *Acinetobacter genomospecies* M1B (99%). While the results of the biochemical test were characterized with very good probability, 16SrRNA analysis revealed that only the last one strain was correctly identified at a species level, and the first two strains were misidentified as Pseudomonadales order. This might be due to limitations of the biochemical identification systems, including Vitek 2, resulting from unequal reproducibility of the test, but also variations in bacterial metabolic phenotype, which is not stable in the constantly changing environment (Tang et al. 1998; Galar et al. 2013). The correct identification of the analyzed bacteria is *Raoultella planticola* M01, and *Acinetobacter calcoaceticus* M1B.

Table 1. The most differentiating biochemical tests result for the three tested strains: *Raoultella planticola* (M01) and *Acinetobacter calcoaceticus* (M1B), obtained with the use of Vitek 2® system.

| **Substance** | ***M01*** | ***M1B*** | **Substance** | ***M01*** | ***M1B*** |
| --- | --- | --- | --- | --- | --- |
| ProA | **+** | **-** | CIT | **-** | **+** |
| ILATk | **+** | **+** | IHISa | **-** | **+** |
| GlyA | **+** | **-** | dCEL | **-** | **+** |
| O129R | **+** | **+** | GGT | **+** | **-** |
| dMAL | **-** | **-** | CMT | **+** | **+** |
| SUCT | **+** | **-** | ILATa | **-** | **-** |
| IMLTa | **-** | **-** | BAIap | **+** | **-** |
| dMNE | **-** | **+** |  |  |  |

ProA - L-prolino-arylamidase; ILATk – L-lactate alkalinisation; GlyA – Glycine arylamidase;
O129R – O/129 resisitance (comp. vibrio); dMAL – D-maltose; SUCT – succinate alkalinisation;
IMLTa – L-malate assimilation; dMNE – D-mannitol; CIT – citrate (sodium); IHISa – L-histidine assimilation; dCEL – D-cellobiose; GGT – *γ*-glutamyl-transferase; CMT - coumarate; ILATa – L-lactate assimilation; BAIap – *β*-alanine arylamidase pNA
